# An approach to classifying sequence tags sampled from *Plasmodium falciparum var* genes

**DOI:** 10.1016/j.molbiopara.2007.03.011

**Published:** 2007-07

**Authors:** Peter C. Bull, Sue Kyes, Caroline O. Buckee, Jacqui Montgomery, Moses M. Kortok, Chris I. Newbold, Kevin Marsh

**Affiliations:** a Kenya Medical Research Institute Centre for Geographic Medicine Research, Coast, Kilifi, Kenya; b Nuffield Department of Clinical Medicine, University of Oxford, John Radcliffe Hospital, Oxford, UK; c Malawi-Liverpool-Wellcome Trust Clinical Research Programme, Blantyre, Malawi; d Department of Zoology, University of Oxford, UK

**Keywords:** PfEMP1, *Plasmodium falciparum* erythrocyte membrane protein 1, DBL, Duffy binding like, PoLV, position of limited variability, DSID, distinct sequence identifier, Malaria, PfEMP1, var, *Plasmodium falciparum*

*Plasmodium falciparum* erythrocyte membrane protein 1 (PfEMP1) appears to play an key role as both a virulence factor and as a target of naturally acquired immunity [1,2]. This large family of molecules is encoded by the highly polymorphic superfamily of *var* genes of which there are 60 variants in every genome [3].

A rapidly growing collection of *var* sequences is now available from clinical isolates around the world [4–11]. Despite immense diversity both in terms of overall organization and primary sequence, the majority of *var* genes contain a DBL1α region [3]. The existence of short islands of homology within this region has enabled the design of primers that can be used to sample sequence from most *var* genes to create DBL1α sequence tags [5]. A standard approach to classification of these sequence tags would enable direct comparisons to be made between different studies. However, the extreme diversity of *var* genes and the fact that they undergo intra-genic recombination [4,12,13], makes this difficult.

Despite the high diversity there does appear to be underlying simplicity to the *var* genes that supports the use of information present in DBL1α sequence tags in making comparisons between the expression levels in different isolates. Analysis of the fully sequenced genome of a single *P. falciparum* isolate 3D7 suggests that the genomic location of the 60 *var* genes promotes genetic structuring and the maintenance of genetically distinct sequence types [14–16]. In addition, structural features of the genes within the single genome of 3D7 closely mirrors the range of structural features among collections of DBL1α sequence tags from clinical parasite isolates [9]. We previously used a small number of key sequence features in an algorithm to classify the DBL1α sequence tags from a single geographical location in Kenya into six groups [9] (see Fig. 1A and below). This *var* tag grouping system, though it is based on portion of the DBL1α domain (see Supplementary information), corresponded well with whole *var* gene classification based on the whole genome sequence of the parasite line 3d7 [9]. This grouping system appears to be biologically meaningful. Expression of group 2 sequences was strongly associated with the parasite rosetting phenotype in Kilifi whereas expression of group 1 sequences was negatively associated with the repertoire of antibodies to infected erythrocyte surface antigens carried by the patient at the time of disease [9]. Thus DBL1α sequence tags appear to contain useful information about the genes to which they belong that is currently not directly accessible in field studies of clinical parasite isolates.

We have developed a rapid approach to performing the classification using text string analysis functions in Microsoft Excel and Perl (see Supplementary files). This classifies sequence tags directly without the need for prior alignment and can be performed on many sequences simultaneously. The approach is summarized in Fig. 1A. The classification is based around a count of the number of cysteine residues within the tag region and a set of sequence motifs at four positions of limited variability (PoLV 1–4) whose positions within the sequence are fixed in relation to four anchor points (a–d, marked with arrows in Fig. 1A). Thus PoLV1 and PoLV4 are fixed in relation to the 5′ and 3′ ends of the sequence, respectively (anchor points a and d). PoLV2 and PoLV3 are fixed in relation to a “WW” motif (anchor point b). The definition of the groups defined by these features is summarized in the box in Fig. 1. Henceforth we will refer to these groupings as cyteine/PoLV groups.

This text string analysis approach was tested on the original set of sequences from Kilifi, Kenya [9] and sequences from 9 other studies (see Fig. 1B–E). The sequences were pre-screened to ensure that they contained a 5′DIGDI and 3′PQFLR consensus sequences. Overall 99.6% of sequences could be classified using this approach. This included 100% of sequences from Malawi (J. Montgomery unpublished), Papua New Guinea [7,17], Mali [10], Solomon Islands [7], and The Philippines [7] together with 100% of sequences from one dataset from Brazil [6]. A dataset from Venezuela (52 non-identical sequences [8]) carried two sequences that could not be classified. A dataset from Brazil (137 non-identical sequences, [18]) carried one sequence that could not be classified. The original dataset from Kilifi (878 non-identical sequences [9]) carried two sequences that could not be classified. All five of these sequences lacked WW or VW motifs required as anchor points within the sequence.

Part of the rationale for this grouping system came from a search for PoLV motifs that were associated with sequences with distinct length distributions [9]. Two motifs were identified which were independently associated with short sequences. These are MFK* at PoLV1 and *REY at PoLV2 (an asterisk here denotes any amino acid). We hypothesised that if sequences of different length recombine with each other they will generate a wide range of sequences of different lengths whereas genetically isolated sequences, i.e., those that are not recombining with one another are able to maintain distinct distributions in their length. If these groupings are genuine the sequences classified into different groups should have similar lengths in different settings. As shown in Fig. 1B–E, broadly similar distributions of sequence length are observed within the six different groups between three different continents, suggesting that sequences generated in these different studies shared the same set of structural features. Specifically, MFK* (carried at PoLV1 in group 1) and *REY (carried at PoLV2 in groups 2 and 5) are associated with short sequences in each geographical region. No examples of sequences with both MFK* and *REY motifs were found, suggesting that these motifs are mutually exclusive. In addition, though *REY motifs were found in sequences with 2 or 4 cysteine residues (cys2 or cys4), with the exception of a single cys4 (group 4) sequence from the Philippines, MFK* motifs were found exclusively in cys2 (group 1) sequences.

Further support for the cysteine/PoLV groupings comes from recent publications. Trimnell et al. found a good correspondence between cysteine/PoLV groupings of cys2 sequences and groups defined phylogenetically within a globally sampled subset of *var* genes with a specific upstream control region, upsA [11]. Also evident from sequences reported in that study is the fact that DBL1 from two other globally sampled subsets of *var* genes can be easily distinguished from DBL1 domains from other *var*s using unique PoLV motifs. *var*2csa *vars* have a unique PoLV2 motif “EVIT”, whereas Type3 *var*s have a unique PoLV4 motif “PPVV” (data not shown).

Kraemer et al. have recently performed an analysis and re-classification of whole *var* genes from 3D7, HB3 and IT4 [19]. Fig. 2A and B summarizes the relationship between the cysteine/PoLV groupings and whole *var* gene classification. With the exception of group 6 sequences which were not found in HB3 *var* genes all sequence groups were represented. In all three genomes cysteine/PoLV group 1 sequences are exclusively found in group A *var* gene and long genes with >5 domains whereas cysteine/PoLV group 5 are found only in non-group A genes and those with 4–5 domains. Cys2 sequence tags (groups 1–3) were never found in group C *var* genes.

Kyriacou et al. used a phylogenetic approach to compare DBL1α sequence tags from Mali [10]. Visual inspection of the layout of these sequences reveals three main groups and a minor group. There was good correspondence between these groups and the cysteine/PoLV groupings (Fig. 2C [10]). This study showed that cys2 sequence tags were more frequent among parasite isolated from children with cerebral malaria than those from children with hyperparasitaemia. However, division of the sequences into cysteine/PoLV groups suggests that the frequency of group 2 sequences is similar in parasites from these two groups of children (Fig. 2D [10]).

At a higher level of resolution, the distinct sequence identifier (DSID) (see Fig. 1A) is a potentially useful method of further classifying sequence tags. This consists of a string of sequence features in the form “PoLV1-PoLV2-PoLV3-number of cysteines-PoLV4-sequence tag length”. The DSID captures more of the overall sequence diversity than the previously described “sequence signature” [9] whilst remaining robust to minor changes introduced by sequencing or PCR errors. Among the 1595 non-identical sequences identified in all the studies described here, there were 1111 DSIDs. Fig. 1F–G illustrates the potential usefulness of this approach to classification. In Fig. 1F, 44 “common” sequences that were shared between more than one study were selected. Fishers exact test was used to determine whether these common sequences were shared between two studies more or less than would be expected by chance (+ or − symbols, respectively). Fig. 1G is the same except that the analysis was done at the level of 157 “common” DSIDs that were shared between more than one study. In contrast to Fig. 1F, there was a highly significant similarity between *var* genes from South American isolates in support a recent study of Amazonian isolates [18]. In contrast to the low overlap between DSIDs from Kilifi and from South America (Fig. 1G) there is considerable overlap in the constituent PoLV motifs themselves (see Supplementary information). This illustrates the potential for recombination to generate diversity from a limited number of sequence blocks [4,12,13].

Since the cysteine/PoLV system of classification is based on commonly occurring sequence features it is hoped that it will useful for initial analysis and annotation, comparison of different geographical regions over time and identification of unusual sequences.

## Figures and Tables

**Fig. 1:**
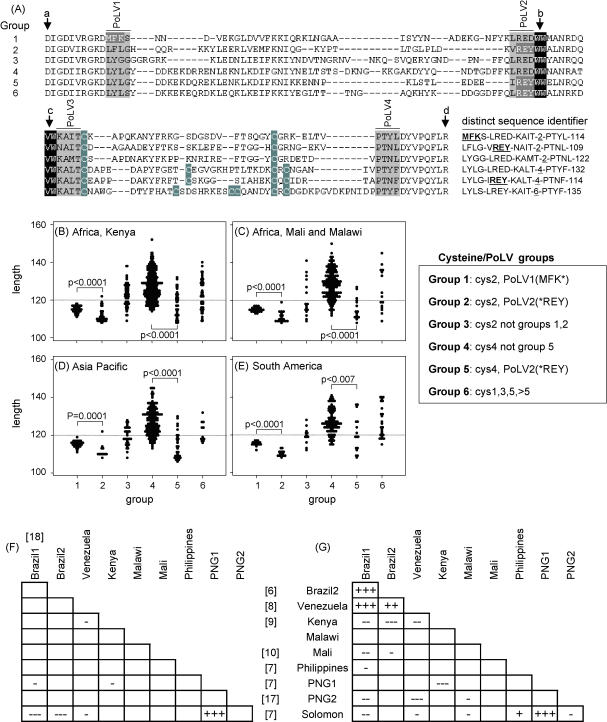
The cysteine/PoLV classification approach. (A) Sequence features extracted from DBL1α sequence tags. The input sequence is the DBL1α sequence starting from a DIGDI motif within homology block D and ending in PQFLR motif within homology block H (see Ref. [20]). Three features are used to group the sequences. These are (1) the PoLV1 motif situated at the 3′ end of homology block H (defined as the four amino acids starting 10 amino acids 3′ to the beginning of the DIGDI consensus), (2) the PoLV2 motif situated at the 5′ end of homology block F (defined as the four amino acids starting four amino acids 5′ to anchor point b or 12 amino acids 5′ to anchor point c, anchor points are marked with arrows), and (3) a count of the number of cysteine residues within the sequence. Two conserved internal anchor motifs “WW” and “VW” (anchor points b and c, respectively) were used to identify homology block F. The “WW” motif was confirmed to be present no more than once within all DBL1α sequence tags analysed. In sequences where the “WW” motif is absent due to sequencing or PCR errors the “VW” motif (anchor point c) is used as a backup. Groups were defined as described previously [9] (see box (*) any amino acid). The “distinct sequence identifier” DSID is defined as “PoLV1-PoLV2-PoLV3-number of cysteine residues in the sequence-PoLV4-sequence length”. (B–E) Length comparisons of sequences from different groups of sequence tags. The lengths of the sequence tags classified into different groups were compared between 10 studies. The studies were classified into four groups (a) Kenyan sequences from Kilifi, (b) non-Kenyan African sequences, (c) Asia Pacific sequences, and (d) South American sequences. The dotted line is placed to aid comparisons of sequence lengths (set at 120 amino acids). To avoid inclusion of the same sequence twice with minor differences due to PCR or sequencing errors, only “distinct” sequences were used from each of the 10 studies (i.e., only one sequence was included for each DSID counted within each individual study, see A). The number of distinct sequences are as follows: (a) *Kenyan sequences*: 606 sequences from Kilifi Kenya [9], (b) *Other African sequences*: 108 from Malawi (Montgomery, unpublished), 124 sequences from Mali [10], (c) *Asia Pacific sequences*: 162 from Papua New Guinea [7,17], 70 from The Solomon Islands [7], 53 from The Philippines [7], and (d) *South American sequences*: 49 from Venezuela [8], 148 from Brazil [6,18]. Example *P* values in comparisons of sequence length between groups are shown (Mann–Whitney *U*-test performed using Stata, Stata Corp, Texas, USA). (F–G) Use of full sequence tag identity and DSID identity to compare sequence overlap between different studies. Only “common” sequence tags (*n* = 44) or DSIDs (*n* = 157) that were shared between more than one study were included in the analysis. Fishers exact test (using Stata) was used to calculate a two sided *P*-value for the distribution of sequence tags or DSIDs between each pair of studies. (+) A significantly more shared sequence tags or DSIDs than would be expected by chance between a pair of studies and (−) significantly less than by chance. +++/−−−, *P* > 0.001; ++/−−, *P* < 0.01; +/−, *P* < 0.05. Only +++/−−− scores are significant after Bonferroni correction for multiple comparisons. The reference number for each study is indicated in square brackets.

**Fig. 2:**
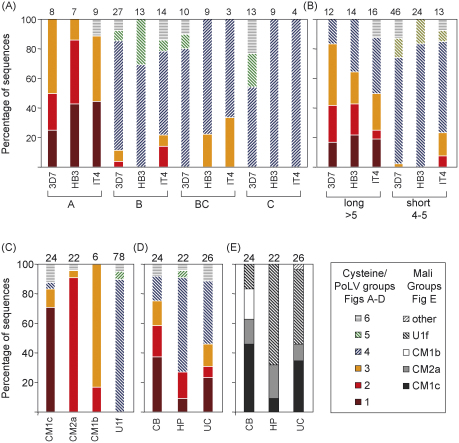
The relationship between the cysteine/PoLV classification approach and other *var* gene classifications. (A–B) Comparison with whole *var* gene classification in laboratory isolates 3D7, HB3 and IT4 [19]. (A) The relationship between whole gene classifications (groups A,B,BC,C) and the cysteine/PoLV classification of the tag region. Group BC comprises the subgroups B1C-B4C defined by Kraemer et al. [19]. (B) Comparison with a crude classification of the full length genes based on the number of domains they contain (>5 or 4–5). *var*2csa and Type3 *var*s are excluded from this analysis because their DBL1 sequences can be clearly distinguished from other *var*s (see text). Comparisons with phylogenetic analysis of sequences from Mali [10]. These “Mali groups” are given names based on a representative member of each phylogenetic group: CM1c, CM2a, CM1b, U1f. (C) Comparison of cysteine/PoLV groups with Mali groups. Distribution of sequence tags from clones picked at random from parasite cDNA libraries from three categories of malaria patients. Proportions of cDNA sequences falling in each group are shown, counting each individual sequence only once for each patient. A maximum of three dominant sequences from each cDNA library (i.e., each parasite isolate) are considered. CB, cerebral malaria; HP, hyperparasitaemia; UC, uncomplicated malaria. (D) Sequences are grouped by cysteine/PoLV groups. (E) Sequences are grouped by Mali groups. [*Note*: a classification system has previously been suggested for DBL1α domains based on phylogenetic comparison of whole DBL1α domain sequences. DBL1α domains were classified as either DBL1α or DBL1α1[15]. DBL1α1 sequence tend to have two cysteine residues within the DBL1α sequence tag region and in this respect correspond with groups 1–3 of our grouping system. However, the correspondence is not exact. Several examples of groups 1–3 sequences can be found in a recent study [19] which are classified as DBL1α rather than DBL1α1 (see Supplementary information). Visual inspection of such sequences suggests the existence of chimeric DBL1α/DBL1α1 domains (for example PFF0010w and PF08_0140 in 3d7). The existence of mosaic domains highlights the need for a strictly defined classification that is specific to DBL1α sequence tag regions that are sampled in field studies]. Numbers of sequences in each comparison are shown above each column.
