# Supplementary material for: An approach to classifying sequence tags sampled from Plasmodium falciparum var genes
Source: Mol Biochem Parasitol. 2007 Jul;154(1):98–102. doi: 10.1016/j.molbiopara.2007.03.011 (PMC1906845; doi:10.1016/j.molbiopara.2007.03.011)
Supplement: Supplementary file 1 [file mmc1.zip › Supplementary information.doc]

**Supplementary Information**

**1) Instructions for the use of the classification algorithm**

Two approaches are presented. One uses Microsoft EXEL spreadsheet functions (sequenceClassifier3.xls). The other uses Perl (sequenceClassifier3.pl)

***EXEL file***

1) Prepare sequences in an EXEL file with the name of the sequence in the first column and the sequence in the second column starting an ending at the same positions as the sequences in Figure 1A.
2) Paste the 2 columns into the first 2 columns of file "sequenceClassifier3.xls"
3) Group classification appears in column I distinct sequence ID appears in column K

***Perl script***

1) Prepare a fasta file of the sequences to be classified, starting an ending at the same positions as the sequences in Figure 1A.
2) Open file "sequenceClassifier3.pl" in the same folder
3) Enter file name of the sequence file when requested

**2) Sequence tags from DBL domains classified as “DBLα” with “DBLα1” like features within the tag region.**

A list of sequences is given from IT4, HB3 and 3D7 isolates. For these, the full var sequence is available whereas the DBL1α tag sampled from field isolates is only a portion of a domain (see figure). The existence of such sequences highlights the need for a classification of tag sequences that is independent of the DBLα and DBLα1 system. DBLα1 like features are in bold and underlined. DBLα like features are in blue and in parentheses. For each sequence the cysteine/PoLV group is indicated

**IT4**

IT4var11 DBLalpha (group 2)

DIGDIIRGKDLFLGHKQRKNQLEERLKTMFANIQNENQDLTTLKPEEVREYWWALNRDQVWKAITCD**A**GVKDTYSINTGNGITSFDFK**CG**HEDKNVAT**NL**DYVPQ**(Y)**LR

IT4var13 (group 2)

DIGDIIRGKDLFLGHQQRKKYLEERLEKMFENIKNNNEYKLGHLSTEKVREYWWALNRQQVWNAITCN**A**EGNDKYFKKSSGGEYKFSGGK**CG**HNDDNVPT**NL**DYVPQYLRW

ITvar29 (group 3)

DIGDIVRGKDLFLGHKQRKKELEENLRNIFKNIHGGLTNGAEARYNDDTGDFFKLREDWWNANRATVWKAITCR**A**GENDTYSINTGNGKMVFWNYK**CG**HNETNVPT**NL**DYVPQFLR

ITvar20 (group 3)

DIGDIVRGKDLYLGYDDEEKEKRKQLEKNLKKFFQKIHDDVMKTSGRTNGKKSAEAQKRYNDATGNYYKLREDWWNANRDQVWKAITCD**A**ADNDEYFENSSDGLYVFSNGQ**CG**RNEGKVPT**NL**DYVPQHLR

**HB3**

HB3var17 (group 3)

DIGDIIRGKDLFIGYNEKDREEKKQLQDNLKDIFKKIYEELSRTNGEAAKKHYEDKDTDKNFFQLREDWWALNRDQVWKAITCS**A**GENDIYSKTTDNGKTTVLYYK**CG**HEDENVAT**NL**DYVPQFLR

HB3var21 (group 3)

DIGDIIRGKDLFIGYNEKDKEEKKQLQDSLKNIFAKIHSEVTKGGNNAEELKARYKEDPNKNYYQLREDWWNANRQEVWRAITCG**A**GENDIYSKTITYGTTTTSNPR**CG**HEDQDVQT**(Y)L**DYVPQ**(Y)**LR

**3D7**

PFF0010w (group 2)

DIGDIVRGKDLFLGHQQRKRKLEENLKQMFENIKKNNDKLDKISIEQVREYWWNANRDQVWEAITCH**A**AHSDEYFRKSTDGVTLYFDGR**CG**RELSSVPT**(Y)L**DYVPQFLR

PF08_0140 (group 3)

DIGDIVRGKDLYLGDQQEKLYLENNLKKIFAKIHENLNDKIKSNYNDSEGNYFKLREDWWTANRDQVWKAITCN**A**PKDANYFEYNSGKFFKFSNDQ**CG**HNNGGAPPT**NL**DYVPQFVR

PFL0005w (group 3)

DIGDIVRGRDLYRGNNRENDKLEKKLKGYFKKIYDNLVEKKKEEAETDYKDDAPDFYQLREDWWALNRQDVWKAITCD**A**HDSRYRKMGADGSITESAMRQ**C(R)**NVADVPT**N(F)**DYVPQ**(Y)**LR

**3) Distribution of PoLV in Kilifi and South American sequences**

78 DSIDs from Kilifi and 25 from South America were selected that were 1) identified in more than one of the 10 studies (i.e. the same DSIDs as used in Figure 1 G) but 2) not present in both Kilifi and South America. The percentage of DSIDs containing each PoLV motif is shown for both sets of sequences. Despite non-overlapping DSID’s there was still considerable overlap in the constituent PoLV sequences suggesting that differences between sequences DSIDs in the two regions is due largely to the combination of PoLV motifs present rather than the PoLV motifs themselves. In support of this idea, the finding of highly significant overlap between DSIDs from three South American studies and lack of overlap with Kenya was found when analysis was repeated using DSIDs with the tag length data removed, leaving the previously described sequence signature [9] (data not shown). Asterisks mark the PoLVs present in DSIDs identified in all three South American studies but absent from Kilifi, showing a bias away from the most common PoLV1 and PoLV3. Though not present in the 78 Kilifi sequences analysed here, the LYIG at PoLV1 was present at low frequency within the complete Kilifi dataset. In contrast the LRET motif at PoLV2 was found in the three South American studies but in none of the other seven studies.
